# What Is Quackery? And Who Art Quacks?

**Published:** 1860-01

**Authors:** J. L. Levison

**Affiliations:** 7 Wentredge Villas, St. John's Wood, London


### ARTICLE III.

### *What is Quackery? And who art Quacks?*

*To the Editors of the American Journal of Dental Science.*

"Is there a hole in a' your coats,
I rede ye tent it,
A chiel amang you taking notes
And faith he'll prent it."—*Burns.*

SIRS:—Though so much has been written to elucidate these questions, there is still great ambiguity in their use.

Having an intuitive dislike to quackery of all kinds, from the immoral consequence, which result either to the actors or their dupes, it is my intention to add a few thoughts on this important subject. But in a paper of this kind, I shall not enter into the peculiar mental constitutions

"Of the cheated and the cheats,"

merely remarking, *en passant*, that the predisposing tendency in professors of this class is excessive cupidity and deficient conscientiousness, whilst most of their victims possess large *marvelousness* which is stimulated into a feverish activity by the extravagant boasting of those who intend to profit by their credulity and if these unreflective believers think at all, it is evident they conclude "that where there is so much smoke—there must be some fire." Yet in order that the reader may understand the sense in which these terms are used, the definition is simply that "Either some great *profession* is made which the boaster knows can never be realized. Or else the quack is so stolidly ignorant as to be incapable of distinguishing the difference between the accidental and the consequential, for as he is without any theory or positive knowledge on the subject he professes to treat, he is liable to mistake some fortuitous result in reference to some special case as applicable to all others, even when positively dissimilar, and that without reflecting on the difference in age, sex, and temperament, which in medicine (as with food) requires some modification in the treatment.

Thus the charlatan acts without any real knowledge, and at variance of the experience of the studious observer of diseases and their phenonomena. And when he dispenses his *specific*, it is to gratify his inordinate avarice, even if it proves injurious to his confiding victims. And though he may become cognizant of the fact, still he continues with unblushing impudence his unprincipled conduct. He is both a cunning and mendacious personage, whether he has a degree indicating his positive or superlative ignorance—yet if he wears this *badge* of his order, he exults in his infamy, though good men shun and execrate him.

There have been, however, many highly gifted men who cured diseases *empirically* at all periods of European history, who had never devoted themselves to the special study of the physical sciences or natural history, and who yet observed many facts in both. These self-taught students were *not* quacks :—their college was the vast temple of the living God! Their books were the different pages of the sublime volume of the universe. Their *curriculum* embraced clear observations on all phenomena affecting health and disease, which are in common parlance called the "laws of nature," without having in all human probability, learned the technical phrases to indicate the difference of structure which are patent in both states, but which still they mentally distinguish.

In modern times, there are some men who have obtained a rank in science by their own labor and diligence; and whose lives have shown, that they had been impressed with the *dignity* of knowledge, and have preferred poverty with rectitude, rather than purchase rank or wealth by any compromise of their moral consistency. Surely, none of the latter can be consided quacks.

On the other hand, are there not many who have had every advantage, and who nominally assume the rank of surgeon or physician? having luckily passed from some *laxity* or favoritism of the examiners, who ought to have regarded them as absolutely unqualified for such a profession. Their practice must therefore be of a mere routine kind—the disease and the dose—and they are likely to misunderstand the one and overdo the other. And all these dangers result just because they are incapable of distinguishing between the symptoms and the causes! How should they, when they have never consumed "the midnight oil," or read a single leaf of the *arcana* of nature. They are satisfied that *their* road to success does not depend on knowledge, but upon the meretricious circumstance of having a good connection or *plenty* of money. And if they may not have the last solid qualification—they may be patronized by some twig or branch of the aristocracy.

*Quackery* is therefore what I have defined it to be, the offspring of ignorance and cupidity. But in saying this, it is essential to distinguish between positive ignorance, which is owing to an insufficiency of all real knowledge, and which might have been obtained by ordinary industry, and that *negative* ignorance induced by the almost inseparable difficulty of investigating the processes carried on in "nature's laboratory," in mysterious silence, and which, may be, depend on causes undefined and thus designated "occult."

Unless some such broad distinction is made, the term quackery might be applied to everything, of which all that was known of it, was derived from mere experience? For the term *quackery* and *empiricism,* are used synonymously, as expressing one condition. But this would be a too sweeping definition, and would implicate every regular professor of medicine. For if any one of the most gifted practitioners were asked to give the *modus operandi* of calomel, and why it acts on the liver and affects its secretions? He could only answer, "we know it by experience." The same might be said, if the question referred to the sedative effects of opium—he could but say, "we know it is so!" and so forth.

And, as the word SCIENCE means simply knowledge, yet, it is knowledge arranged and systematised. And this very collocation explains that its details are obtained from different observers. Therefore, for all practical purposes, it may fairly be said "that *quackery* should be applied to extravagant promises, which it is impossible to realize."

In dental practice this is a well-marked distinction—for pledges are made of doing wonderful things with a consciousness of the absurdity of such mendacious statements, and which the really scientific never are guilty of making.

The scientific dentist, like the medical practitioner, knows that different persons require particular treatment; and that there are particular idiosyncrasies which form altogether exceptional cases. Like true modest men of science, they know how much of obscurity exists in many special diseases, and hence they never speak of anything as a specific. In toothache, for example, what is a "cure-all?" Turpentine will allay the *neuralgic* pain in one, and drive another nearly mad. The same may be said of all the other "remedies."

Although it is then justifiable to state what science and experience teach, but still, the more any one knows, the more cautious he is not to promise extravagantly. "He will try to cure." "He hopes to relieve." "He thinks it is very likely that the patient will soon be free from pain." &c.

The quack does just the contrary—he speaks with a bombastic certainty, strokes his beard, as if to draw inspiration from it and not his brain, and swaggers about his great acquirements and his superlative superiority, and all in the actual ratio of his profound ignorance. And in so doing he excites the fears of his dupes and succeeds in "putting money in his purse." He is too obtuse to care for "character," and he is satisfied even if his victims *curse* him "not loud but deep," so that he can rattle his pocket and feel their *golden* contributions. The quack has therefore no subsequent misgivings, and the only thing that disturbs his self-complacency is "the fear of the law," and the dread that he will have to refund his ill gotten and extravagant fees.

Should his victims make no attempt to expose and punish him, he goes on with greater recklessness, and every subsequent imposition deadens the little *morsel* of sense he may have had when he entered on his unprincipled vocation. He fattens on the ignorance and credulity of those whom he, spider like, drags into the meshes formed by his cunning and avarice, and he then will, without remorse, squeeze from them their last guinea, with as little compunction as that sanguinary insect does to the last drop of blood of some silly, thoughtless fly caught in his web.

Two modes of prevention may be indicated, not only to put an end to the immorality of quacks, and the suffering and disappointment of their victims, which are simply the following:

1st. By means of making physiology a part of the education of all classes. Just enough, by means of models, that they may learn the marvellous structure and complexity of their frames—the seat of the vital organs, and a popular exposition of digestion, nutrition and assimilation, &c.

If such were the case, and any symptom of disease afterwards was manifested, it is improbable that any ignorant charlatan would be applied to, but only such practitioners as were well acquainted with the mechanism of the human body.

2d. In all civilized countries no one should be allowed to practice (under the liability of severe penalties) if he had not undergone a rigid examination as to his theoretical and practical knowledge, where especially, as in dentistry, proficiency in the latter is so imperative for the comfort of his patients.*

In the absence of these distinctive and conservative arrangements, it should be decided to place any one convicted of quackery in a sort of "MORAL PILLORY," (without using the offensive missiles used in the ordinary one,) but the delinquent should be attacked most pungently with the hope that "the still small voice" might awake in him some remorse for his misdeeds, and make him turn from his evil ways.

If all these means are neglected, quackery will and must continue, and the repetition of the acts of the dishonest will tend to render callous the consciences of the professors of this "black art," and they will continue to enjoy their ill-gotten wealth, because those victimized give them an impunity from a false delicacy. Thus they not only do actual injury to those whom they have cheated, but they indirectly injure respectable and upright men practicing in the same special practice. In the profession of dentistry this

* Candidates for membership of the College of Surgeon Dentists in England were received without any examination, either anatomical, physiological, pathological, or mechanical.

is notorious. The people who have suffered from the ill-treatment of unqualified persons form a bad opinion of the art itself, and regard all belonging to this branch of practice as not over scrupulous. The *few* who are regarded as probably *exceptions*, are those who get the "lion's share" of patronage among the most educated classes.

If you think these remarks worthy a place in your excellent Journal, I shall be obliged by your inserting them.

I am, sirs,

Yours, respectfully,

J. L. LEVISON.

**7 Wentredge Villas, St. John's Wood, London, Aug. 1st, 1859.**

